# Use of a Spectrophotometric Method for the Detection of Adulterants in Commercial Fulvic Acid Products

**DOI:** 10.1093/jaoacint/qsaf073

**Published:** 2025-08-13

**Authors:** Elena A Vialykh, Shelby Buckley, Julia Gentile, Fernando L Rosario-Ortiz, Richard T Lamar, Jarrod Psutka, Mohammad Rahbari

**Affiliations:** University of Colorado Boulder, Department of Civil, Environmental, and Architectural Engineering, Boulder, CO 80303, USA; Perm National Research Polytechnic University, Faculty of Chemical Technologies, Industrial Ecology and Biotechnology, Perm 614990, Russia; University of Colorado Boulder, Department of Civil, Environmental, and Architectural Engineering, Boulder, CO 80303, USA; University of Colorado Boulder, Department of Civil, Environmental, and Architectural Engineering, Boulder, CO 80303, USA; University of Colorado Boulder, Department of Civil, Environmental, and Architectural Engineering, Boulder, CO 80303, USA; University of Colorado Boulder, Environmental Engineering Program, Boulder, CO 80303, USA; Huma Inc, Gilbert, AZ 85233, USA; BioLiNE Corporation, Alvinston, ON N0N-1A0, Canada; BioLiNE Corporation, Alvinston, ON N0N-1A0, Canada

## Abstract

**Background:**

Numerous products manufactured from non-humic sources have flooded the market claiming to be fulvic acids. The challenge is finding an easy method to distinguish between products containing genuine fulvic fractions and those containing adulterants. UV spectrophotometry has been widely used to study the fulvic fraction extracted from humic substances, with multiple metrics derived from UV absorption spectra developed and implemented by researchers.

**Objective:**

Leverage ten indices that are characteristic features of the UV spectra of hydrophobic fulvic acids to differentiate products containing authentic fulvic fractions from those containing adulterants.

**Methods:**

Fulvic fractions were diluted to 5 ppm carbon and UV spectra were obtained. Spectra were normalized and analyzed to calculate 10 different indices. The percent difference between the index values of the product and the corresponding index values for the Suwannee River fulvic acid (SRFA) and Pahokee peat fulvic acid (PPFA) standards were calculated. An equally weighted average for all 10 indices was calculated and a 70% cutoff value was used for the average percent error as a screening tool to distinguish products containing fulvic fractions from adulterants.

**Results:**

Fifty-four test samples were analyzed, with nine samples being analyzed by two different laboratories using the established method. Fourteen of the 25 commercial products studied were found to contain fulvic fractions. Increased metal ion concentration within the investigated range did not impact the average percent error calculated, nor did varying the total organic carbon concentrations of the test portions within the range of 1–10 ppm.

**Conclusion:**

The method investigated could be a suitable screening tool for most commercial products and is capable of accurately distinguishing products that contain fulvic fractions.

**Highlights:**

The method accurately found all 11 fulvic fractions isolated from known humic substances as fulvic, and all 11 test samples prepared from non-humified materials as non-fulvic.

The utilization of humic substances (HS) as biostimulants in agriculture has gained significant attention in recent years due to their potential to enhance crop productivity and sustainability (1). HS are becoming increasingly popular because of their ability to enhance nutrient uptake, increase abiotic stress resistance, and promote plant growth (2). Numerous commercial products, produced from non-humic sources, have flooded the market claiming to be fulvic acids. However, amidst this proliferation of products, concerns have arisen regarding the authenticity and quality of some products being sold. Currently, a robust method to differentiate adulterant products from authentic HS does not exist. Consequently, there is a pressing need for a robust method to authenticate these products and ensure they are produced from natural humic materials (e.g., peats, oxidized lignites, and sub-bituminous coals).

HS are the products of the breakdown and transformation of dead plant tissues that result from a combination of natural processes including microbiological, faunal, and chemical transformations. HS are extremely heterogeneous, and their properties vary based on source, making the chemical structure of HS diverse. Fourier-transform ion cyclotron resonance mass spectrometry (FTICR-MS) analysis has revealed that humic acids (HAs) and fulvic acids (FAs) are complex mixtures of thousands (e.g., >10 000) of individual molecules that range in molecular weight primarily between 200 and 800 Da (3,4). Representative broad chemical groups include highly condensed aromatic compounds, lignin- and tannin-like compounds, carboxylic-containing aromatic and aliphatic molecules, fatty acids, proteins, and carbohydrates (4).

HAs and FAs are two subfractions of HS, operationally defined based on their solubility in aqueous solutions. HAs are soluble in alkaline solutions but insoluble in acidic solutions, while FAs remain soluble regardless of pH (5). Typically, HS are extracted from sources like compost, peat, or coal using strong alkaline solutions such as NaOH or KOH. Subsequently, they are separated from insoluble materials through centrifugation or settling. To isolate HAs from FAs (referred to as the fulvic fraction, FF), the pH of the alkaline extract is adjusted to pH levels ranging from 1 to 2 using either strong inorganic acids (e.g., hydrochloric acid or phosphoric acid) or organic acids (e.g., citric acid). This pH adjustment leads to the precipitation of HAs, which can then be separated from the FF using filtration or centrifugation. The FF can further be fractionated into hydrophobic FA (HFA) and hydrophilic FA. HFA is the fraction of the FF that binds to a DAX-8 resin (6). FFs are commonly employed alone or in combination with nutrients in agriculture due to their easy solubility and known biostimulant properties (7).

FFs sold commercially originate from a wide range of sources, including compost, peats, Shilajits, humic and fulvic shales, brown coals and oxidized lignites (i.e., Leonardite) and sub-bituminous coals (i.e., humates or humalites). In composts (e.g., vermicompost), microbiological and faunal activity (e.g., earthworms) are primarily responsible for HS production in a process called humification. In peats, bacterial activity under water-logged conditions is the primary driver for HS production in a process called peatification (8). Coals are produced from peats that have been buried under elevated temperatures for millions of years in a chemical/physical process called coalification (9). HS obtained from brown and oxidized lignite and sub-bituminous coals have already undergone peatification prior to initiation of coalification. Many plant extracts, hydrolysates, effluents from food processing, pulp and paper production, digesters, and industrial activities are also loaded with heterogeneous mixtures of organic molecules; however, these molecules have not been transformed through the process of humification (10). FF solutions are pale yellow- to dark orange-colored liquids that are easily mimicked by these other potential adulterants. Lignosulfonates and corn steep liquor are two common examples of products identified as FAs that are not produced from humified source material. What sets FFs apart from these adulterants is that they are the residues of the processes of humification, peatification, and coalification acting on lignocellulosic substrates to produce complex mixtures of thousands of unique molecules. The wide variety of combinations of different lignocellulosic residues, microbial and chemical/physical transformations, climatic conditions, and time produces HS that possess inter- and intra-source chemical diversities that provide a challenge for the development of a standardized authentication method.

Among the multitude of analytical techniques available for HS characterization, UV-Vis spectroscopy stands out as one of the most widely used tools. Absorption spectra were first used as a proxy for the concentration of HS in surface waters (11), and this technique has evolved from a quantitative surrogate for concentration to a qualitative descriptor for physicochemical reactivity and biogeochemical origin. The absorption of light by HS results in a characteristic spectrum, often showing an exponential decay with increasing wavelength. This shape, inherent to HS, originates from its complex mixture of organic compounds, such as HA and FA molecules, and other chromophoric substances. Each component contributes a unique spectral signature, collectively shaping the observed exponential decay in the UV spectrum of HS. In addition, a wide range of metrics derived from the absorption spectra have been developed and implemented by the environmental science and engineering community, such as the spectral slope and specific absorbance. The exponential shape of HS UV-Vis absorption spectra is widely observed regardless of the source and is usually featureless, though some shoulders or peaks can be observed from the presence of ions or metals. By leveraging spectral features characteristic of HFA, the UV-Vis spectrum could be used as a tool to differentiate FFs from adulterant products.

Despite the wide use of UV-Vis spectroscopy to characterize HS, there is a notable absence of published literature describing the qualitative distinction between FF products and potential adulterants found in the market using this technique. Mayhew and co-authors used absorbance values between wavelengths of 290 and 330 nm, in 5 nm increments, to differentiate HS from other non-humified materials (12). The absorbance data were zeroed by subtracting the minimum absorbance in that series from all values, and it was then normalized by dividing the absorbance at the other wavelengths in the range by the absorbance at 290 nm (12). The squared sum of the scaled absorbance difference (SSSAD) was calculated by summing the differences between the sample scaled absorbance and the standard curve scaled absorbance at every 5 nm within the range (12). The approach showed promise; however, the authors did not provide any data on HFA or FF being determined as humified using the method. Rahbari and co-authors used the method (12) to analyze HFA test samples prepared using FA standards from the International Humic Substances Society (IHSS) and FFs extracted from humic sources; the method failed to determine these HFA and FF as humified (10).

The most extensively studied FA is Suwannee River HFA (SRFA), which has been thoroughly analyzed over decades (13). Furthermore, Suwannee River natural organic matter (NOM) and FA standards extracted from other sources are readily accessible from the IHSS, facilitating their widespread use as reference materials. Given its widespread availability and analytical consistency, SRFA was selected as the reference standard HFA in the development of this method and was used for comparison with other HFA and FF samples. A second IHSS reference standard, Pahokee peat HFA (PPFA), was also used in this study to increase the robustness of the method.

This study aimed to build on prior research and to develop a reliable UV-Vis spectroscopy-based method for discriminating between authentic FA products (derived from humified sources) and adulterants. We propose using multiple features of the UV absorbance curve, with the aim of increasing the reliability for accurate differentiation between humified FFs and non-humified substances. Table 1 provides details on the 10 contributing indices selected from literature and used in the analysis for this method. Each index correlates with a specific characteristic of FAs. Thus, we selected indices to comprehensively characterize HFA, FFs, and adulterants. Our objective was to develop an initial screening tool that could be used for products claiming to be FAs.

**Table 1.** Description of indices used for FA characterization calculated from the primary UV-spectra

Index	Determination or calculation	Explanation	Ref.
Specific absorbance	Absorbance coefficients taken at 254, 280, 325, 355, 412, and 440 nm	General characteristics	(5)
a @ 254	UV-absorbance coefficient at 254 nm	Indicator of FA aromaticity; positively correlates with aromatic C content of FAs	(14–16)
a @ 355	UV-absorbance coefficient at 355 nm	Positively correlates with metal ion complexation	(17, 18)
S @ 275, S@350	Spectral slope from 275 to 290 nm, and 350 to 400 nm. S is a measure of how fast the absorption decreases with increasing wavelength. Calculated below where a_λ_ is the absorption coefficient at specific wavelength and λ_0_ is the reference wavelength. K is a background constant $a_{\lambda }=a_{\lambda_{0}}*e^{-S\left(\lambda -\lambda_{0}\right)}+K$	Correlates with the average molecular weight and aromaticity degree of CDOM. Changes in *S* have been associated with a different origin of CDOM (terrestrial or microbial) and with changes in its pool due to production and degradation processes or interactions with metals	(19–23)
SR(275/350)	Spectral slope ratio (*S*R) at intervals 275–295 nm and 350–400 nm	Relates to molecular weight, photochemical and microbial degradation	(19, 24)
a250/a365	Ratio of absorbances at 250 and 365 nm	Correlates with molecular size & aromaticity of FAs	(25)

## Experimental

### Phase 1—Initial Method Development

There were two phases to the research. In the first phase, the method was developed, and in the second phase the method was refined and validated. SRFA Standard III (Cat. No. 3S101F) and PPFA Standard III (Cat. No. 3S103F) were obtained from the IHSS and used as the HFA standards against which the UV absorbances of all test samples were analyzed.

All test samples analyzed in Phase 1 were generated by R.T. Lamar and are annotated with the letter “A” at the front of their assigned test sample name. A total of 40 test samples were analyzed in Phase 1, consisting of 7 full fulvic fraction (AFF) isolates derived from known humic substance sources; 21 commercial fulvic products (ACFPs) consisting of products marketed as FAs based on their labels; 4 samples derived by alkali extraction of Leonardite humic ores that contain both HA and FF (AHSE); 6 non-humified materials (ANHMs); and 2 natural organic matter (ANOM) materials obtained from IHSS. Table 2 provides a complete list of test samples prepared for analysis in Phase 1 of the study, along with associated details and assigned IDs. Depending on the source material different preparation steps were required to generate the test samples.

**Table 2.** List of Phase 1 samples investigated with the MUVI method^a^

Category	Sample name	Sample description
FF	AFF-01	Idaho ore HFA
FF	AFF-02	New Mexico ore FF
FF	AFF-03	Russian peat FF
FF	AFF-04	Utah ore FF
FF	AFF-05	Australian ore FF
FF	AFF-06	Bonaparte WA peat FF
FF	AFF-07	North Dakota ore FF
CFP	ACFP-01	Commercial product #1
CFP	ACFP-02	Commercial product #2
CFP	ACFP-03	Commercial product #3
CFP	ACFP-04	Commercial product #4
CFP	ACFP-05	Commercial product #5
CFP	ACFP-06	Commercial product #6
CFP	ACFP-07	Commercial product #7
CFP	ACFP-08	Commercial product #8
CFP	ACFP-09	Commercial product #9
CFP	ACFP-10	Commercial product #10
CFP	ACFP-11	Commercial product #11
CFP	ACFP-12	Commercial product #12
CFP	ACFP-13	Commercial product #13
CFP	ACFP-14	Commercial product #14
CFP	ACFP-15	Commercial product #15
CFP	ACFP-16	Commercial product #16
CFP	ACFP-17	Commercial product #17
CFP	ACFP-18	Commercial product #18
CFP	ACFP-19	Commercial product #19
CFP	ACFP-20	Commercial product #20
CFP	ACFP-21	Commercial product #21
NHM	ANHM-01	Molasses
NHM	ANHM-02	Acetic acid
NHM	ANHM-03	Citric acid
NHM	ANHM-04	Corn steep liquor
NHM	ANHM-05	Calcium lignosulfonate
NHM	ANHM-06	Plant food protein
HSE	AHSE-01	Humic substance extract #1
HSE	AHSE-02	Humic substance extract #2
HSE	AHSE-03	Humic substance extract #3
HSE	AHSE-04	Humic substance extract #4
NOM	ANOM-01	IHSS IR 110 N UMR NOM
NOM	ANOM-02	IHSS 2R110N SR NOM

a NHMs are commercial products but because we know that these products are non-humified materials, we have separated them from commercial products (CFPs) that are sold as fulvic acids.

- *AHSE-01 to AHSE-04.—*These samples were prepared according to the alkaline extraction outlined in ISO 19822:2018. A representative portion of each source material was crushed into a fine homogeneous powder and screened through a 75 µm sieve. A 2.5 g portion of the sieved material was weighed and transferred to a 1 L Erlenmeyer flask. The flask was placed on a magnetic stirrer with a 7 cm long magnetic stir bar. Under constant stirring, 0.1 M NaOH was added until a final volume of 1 L was achieved. The head space was evacuated with N_2_, the flask was covered with parafilm, and the solution was stirred vigorously for 18 h. The entire contents of the flask were transferred to centrifuge tubes and centrifuged for 30 min at 3900 × *g* to separate any insoluble material from the dissolved materials in the alkaline extract. The alkaline extract was carefully decanted from the centrifuge tubes into a 1 L Erlenmeyer flask and neutralized to a pH of 6.8–7 using HCl.
- *AFF-02 to AFF-07.—*These samples were prepared using the same steps as outlined above to obtain the alkaline extracts; however, they were further acidified rather than neutralized to separate the precipitated HA fraction and obtain the full fulvic fraction. To achieve this, after centrifugation, the supernatant containing the alkaline extract was transferred to a clean 1 L Erlenmeyer flask containing a magnetic stir bar. The insoluble material left at the bottom of the centrifuge tubes was discarded. While gently stirring the solution, the pH of the alkaline extract was decreased to 1.0 ± 0.1 with 6M HCl. The flask was covered with parafilm and allowed to mix for 6 h. The flask was removed from the stirrer and allowed to sit undisturbed for 4 h allowing the flocculated HA to drop out of solution. The extract mixture was further centrifuged at 3900 × *g* for 1 h. The supernatant that contained the FF was carefully decanted from the centrifuge tubes into a clean 1L Erlenmeyer flask ensuring not to include any of the flocculated HA.
- *AFF-01.—*AFF-01 consisted of HFA extracted from Idaho ore. To further separate the HFA from the FF, the same initial steps, outlined above, were followed to obtain the FF. HFA was separated by selective adsorption to DAX-8, a macroporous adsorbent resin, to which HFA binds. A 4 cm × 25 cm glass chromatography column was partially filled with 280 mL acid-regenerated DAX-8 resin soaked in deionized (DI) water. The FF solution was pumped through the top of the column, into an approximately 4 cm deep layer of DI water that covered the resin, using a peristaltic pump set at just enough pressure so that the flow rate was sufficient to completely cover the resin in the column without overflowing the column or allowing the resin to be exposed to air. The column was washed with DI water until two column volumes of water were eluted. The HFA was then desorbed from the resin by back elution with 0.1 M NaOH. The HFA-containing effluent was captured and transferred to sample tubes to be used for further analysis. Complete desorption of HFA was indicated when the absorbance of the effluent was 0.030 at 350 nm using a spectrophotometer or after eluting three column volumes, whichever occurred first.
- *ACFP-03, ACFP-14, ACFP-17, ACFP-21, ANOM-01, and ANOM-02.—*The four commercial products listed (ACFP-03, ACFP-14, ACFP-17, ACFP-21) and the two natural organic matters standards from IHSS (ANOM-01 and ANOM-02) were in water-soluble powder format. For these samples, 5 mg of each sample was weighed and added to 100 mL DI water in a beaker and stirred for 1 h. The solutions were then filtered using 0.45 µm filters.
- *All other ACFP and ANHM samples.—*The remaining 16 ACFPs and the six ANHM products were all source material obtained in liquid format. These liquid samples were simply diluted and filtered through 0.45 µm filters.

Test portions were prepared from every one of the 40 test samples. The carbon concentration of each test portion was determined using an in-line Sievers M5310c Total Organic Carbon (TOC) Analyzer. Each test portion was then diluted to approximately 5 ppm TOC to generate the aliquot used to obtain UV absorbance data. Ultrapure water with a resistivity of 18.2 mΩ generated from a Sartorius Arium Pro DI dispenser was used in this study.

### Phase 1—UV-Vis Analysis

A Cary 4000 UV-Vis spectrophotometer was used to collect the absorbance spectrum of each approximately 5 ppm C aliquot. Prior to analysis, the spectrophotometer was zeroed and baseline-corrected using ultrapure DI water, or buffer in selected cases, and the wavelength range was calibrated using a holmium oxide reference cell. Each aliquot was added to a 1 cm quartz cuvette, and the absorbance was measured between wavelengths of 200 and 800 nm. A standard HFA was analyzed after every 10 measurements to account for instrument variation. To explore concentration effects, five test portions were diluted with DI water to generate aliquots with TOC concentrations of 10, 7.5, 5, 2.5, and 1 ppm, which were then used to obtain UV absorbance data.

### Phase 1—Metal Effects Analysis

To account for the effects of metal concentrations on the UV-absorbance spectra, a variety of metal salt stock solutions, including calcium chloride, magnesium chloride, and aluminum sulfate, were added in concentrations of 1, 2.5, 5, and 10 ppm to test portions containing 5 ppm C of the SRFA standard and then analyzed.

### Phase 1—Metals Ion Analysis

All 40 test portions diluted to 5 mg C per liter, were digested with concentrated nitric acid using a Perkin Elmer Titan MPS microwave digester following EPA Method 3051A. The sample and acid(s) were placed in fluorocarbon polymer (PFA) microwave vessels or vessel liners. Vessels were sealed and heated in the microwave unit for 35 min. After cooling, the vessel contents were centrifuged, diluted to 50 mL, and analyzed by inductively coupled plasma-mass spectrometry (ICP-MS) using a Perkin Elmer NexION ICP-MS system.

### Phase 2—Second Laboratory Method Verification

Nine test samples from Phase 1 were sent from the University of Colorado to a second laboratory for UV absorbance analysis. The test samples were labelled with the measured carbon concentration (TOC). The test samples were diluted with DI water to 5 ppm of C. The DI water was purchased from Sigma Aldrich and had a resistivity of approximately 18 MΩ·cm. There were four AFF, four ACFP and one ANOM test samples analyzed by both laboratories. Each test sample was diluted in triplicate to generate three test portions from which aliquots were drawn for UV absorbance.

In addition, 14 new test samples were prepared in triplicate for the second phase and diluted to 5 ppm C. The new test samples analyzed only in Phase 2 are annotated with the letter “B” in front of their test sample names. These test samples included four BFFs, five BCFPs, four BNHMs and one BHFA. Table 3 provides a list of the test samples prepared for analysis in Phase 2 of the study, along with associated details and assigned IDs. Aliquots of the 5 ppm C test portions were added to a 1 cm quartz cuvette, and the absorbance was measured between wavelengths of 200 and 800 nm.

**Table 3.** List of Phase 2 samples investigated with the MUVI method.

Category	Sample name	Sample description
FF	BFF-01	N.D Leonardite FF
FF	BFF-02	Canadian sphagnum peat FF
FF	BFF-03	Irish moss peat FF
FF	BFF-04	Balkan black peat FF
CFP	BCFP-01	Commercial product #1
CFP	BCFP-02	Commercial product #2
CFP	BCFP-03	Commercial product #3
CFP	BCFP-04	Commercial product #4
CFP	BCFP-05	Commercial product #5
NHM	BNHM-01	Seaweed extract #1
NHM	BNHM-02	Seaweed extract #2
NHM	BNHM-03	Sugarcane molasses
NHM	BNHM-04	Yucca extract
HFA	BHFA-01	IHSS Elliott Soil FA Standard V

### Phase 2—TOC Measurement and UV-Vis Analysis

In Phase 2 of the research, the following equipment was used: Milwaukee Mi 180 pH meter with a range of 0–14 and resolution of 0.01, Denver Instrument Company TR-204 analytical balance with readability to 0.0001 g, Agilent 8453 UV-Vis spectroscopy system (190 to 1100 nm range, 1.0 nm spectral bandwidth/nominal spectral slit width), and a Shimadzu TOC-L to determine TOC of test samples.

### Spectral Analysis for All UV Absorbance Data (Phase 1 and Phase 2)

The approach to spectral analysis was developed during Phase 1 and was used to analyze UV absorbance data collected in both phases of the study. All UV absorbance data was normalized by dividing absorbance at each wavelength between 200 and 800 nm by the maximum absorbance for that test portion. The normalized absorbance was entered into ASFit, and each of the 10 indices provided in Table 1 were calculated. ASFit is an all-inclusive tool for analysis of UV-Vis spectra of colored dissolved organic carbon (DOC) (19). The approach used in this study for UV spectrophotometric-based qualification of HS was named the MUVI (Multi UV-Absorbance Index) method.

## Results and Discussion

### Unique Shape of FA Spectra

**Figure 1.**
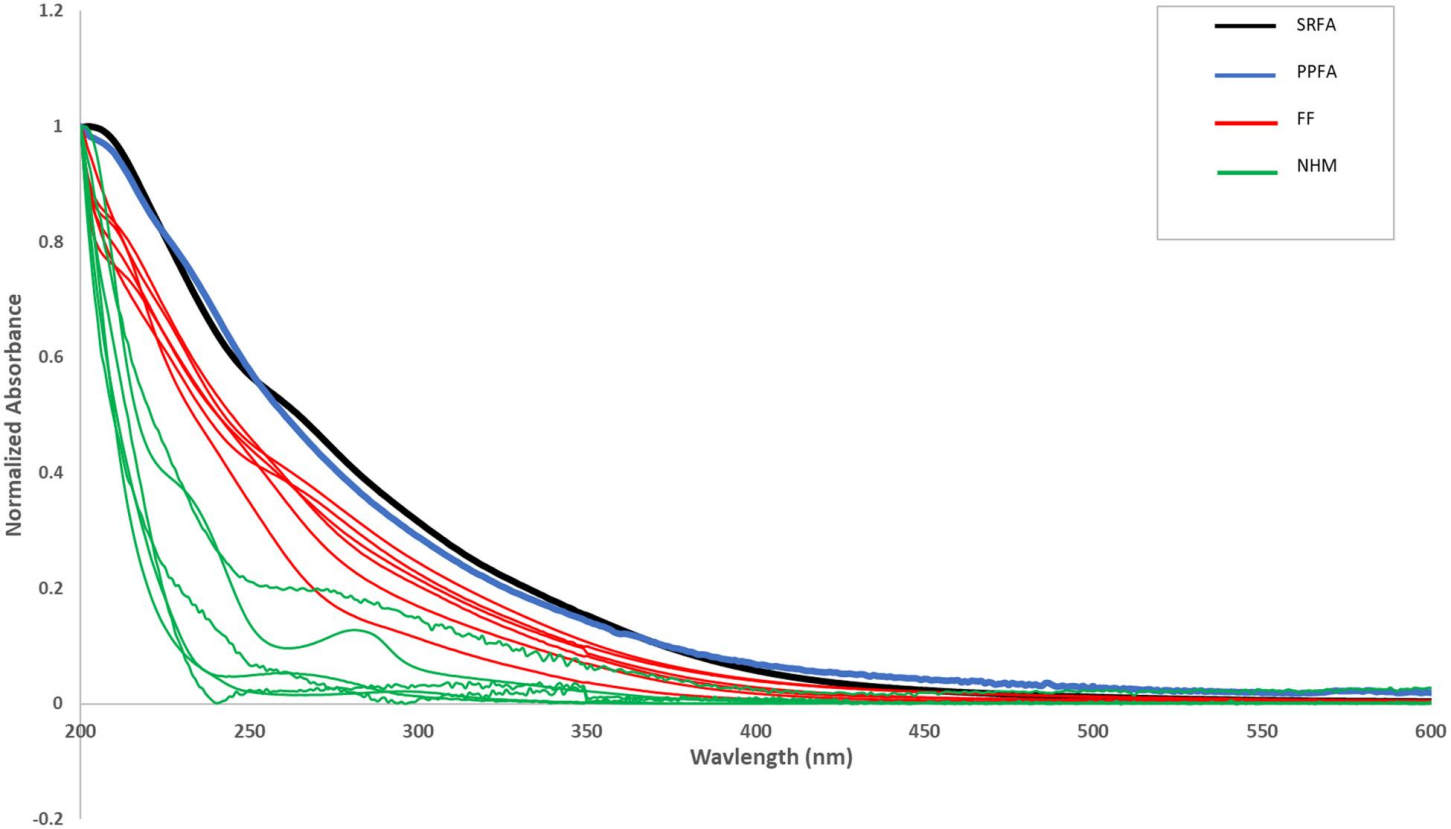
Normalized UV-Vis absorbance spectra for Phase 1 FF and NHM samples.

UV-Vis spectra of HS, including FAs, have a unique shape characterized by an exponential curve with gradually diminishing absorbance as the wavelength increases. The normalized UV absorbance spectra across the entire range studied, comparing the absorbance obtained for the FF and NHM test portions with both the SRFA and PPFA standards, are given in Figure 1. There is separation between the FF absorbance spectra (in red) and the NHM (in green). The SRFA and PPFA were obtained from the IHSS and were used as the HFA standards. The difference in the normalized spectral curve observed between the two HFA standards and the FFs is due to the inclusion of both the hydrophobic and hydrophilic fractions in the FF.

### Development of the Multi-UV Absorbance Index (MUVI) Method

In this study, across the two phases, a total of 54 unique test samples were analyzed. To ensure robustness in the development and validation of the MUVI method, it was important to use many test samples from a diverse range of known FFs, known non-humified materials (NHMs), and commercial products with labels advertising that they contain FAs. The first step in the MUVI method was to examine the raw absorbance measured at 254 nm. If the raw absorbance value of a given test portion was less than 0.08 (the chosen cutoff value, which will be explained below), the sample likely contained some non-humified material and would be deemed “non-fulvic”. Further characterization with fluorescence spectroscopy (excitation emission matrix, EEM), NMR or Fourier-transform ion cyclotron resonance–mass spectrometry (FTICR-MS) would be required to determine whether the product contains some genuine FF. For those samples with raw absorbance greater than 0.08 at 254 nm wavelength, the UV absorbance was normalized by dividing all UV absorbance values for that test portion by the maximum absorbance value of that test portion.

IHSS SRFA and PPFA served as the standards for the MUVI method. The normalized absorbance values for each test portion were entered into AsFit, an all-inclusive tool for analysis of UV-Vis spectra of colored dissolved organic matter (CDOM). This free online tool was used to obtain a calculated value for each of the 10 indices outlined in Table 1. The index values obtained from AsFit were exported to a spreadsheet, and the results were compared independently against the average calculated index values for SRFA and PPFA (the two standards used in the MUVI method). To assess the extent of variation, percent error (PE) was calculated for each index value using


$$\%\;\mathrm{Error}=|\frac{v_{a}-v_{s}}{v_{s}}|*100,$$

where v_a_ = index calculated for the sample and v_s_ = average index calculated for the standard FA. Since there were triplicate test portions prepared for each test sample, each test portion resulted in a PE value calculation for each index. The three resulting PE values calculated were averaged to provide a single PE value for each index for any given test sample, which generated a total of 10 PE values per test sample. The final step in the method was to calculate the overall average percent error (PE_avg_) where each index PE was assigned an equal weight and the final overall PE_avg_ value was calculated across all indices.

The PE calculations across each of the 10 indices for all seven AFF and six ANHM samples analyzed in Phase 1 of the study are given in Table 4. The PEs calculated for each individual index against both the SRFA and PPFA standards are provided along with the overall PE_avg_ calculated for each test sample against both standards. For most of the AFF test samples, the PE_avg_ values calculated against the SRFA and PPFA were relatively close (Table 4). Given the small differences observed in the PE_avg_ values calculated against the two standards, it is recommended that both SRFA and PPFA are used as standards. Rather than averaging the results of the two standards, the lower PE_avg_ value calculated between the two was used in the MUVI method for the interpretation of the data. For some samples that are close to the cutoff, using the lower PE_avg_ value would result in a pass whereas using the higher value would result in a failure.

**Table 4.** Sample percent error calculations for select Phase 1 samples relative to SRFA and PPFA

Sample Name		S @ 275	S @ 350	a @ 254	a @ 280	a @ 325	a @ 355	a @ 412	a @ 440	SR(275/350)	a250/a365	PE_avg_
SRFA	AsFit Indices	0.01	0.02	0.54	0.40	0.22	0.14	0.05	0.03	0.70	4.74	
PPFA	AsFit Indices	0.013	0.016	0.53	0.36	0.16	0.13	0.051	0.03	0.8	4.7	
PE_PP HFA _(%):	SRFA standard	0	11	2	10	27	7	6	0	14	1	7.9
PE_SRFA _(%):	PPFA standard	0	13	2	11	38	8	6	0	13	1	9.0
Percent errors (PE) at each index compared to each standard												
AFF-01	PE_SRFA _(%)	12	25	22	28	34	44	54	56	8	47	32.9
	PE_PPFA_ (%)	12	41	20	20	9	39	57	56	19	48	32.1
AFF-02	PE_SRFA _(%)	24	74	42	61	69	77	88	88	27	211	76.1
	PE_PPFA _(%)	24	96	41	57	57	75	89	88	36	213	77.6
AFF-03	PE_SRFA _(%)	15	1	24	23	30	35	30	21	17	18	21.5
	PE_PPFA _(%)	15	14	23	15	4	30	34	21	3	19	17.7
AFF-04	PE_SRFA _(%)	33	49	26	42	48	55	72	74	8	86	49.2
	PE_PPFA _(%)	32	68	24	35	28	52	73	74	20	88	49.4
AFF-05	PE_SRFA _(%)	9	17	20	27	27	31	40	43	4	22	23.9
	PE_PPFA _(%)	9	32	18	18	0	26	44	43	16	23	22.8
AFF-06	PE_SRFA _(%)	8	8	11	10	15	24	23	17	3	21	13.9
	PE_PPFA _(%)	8	22	9	0	16	18	27	17	10	22	14.9
AFF-07	PE_SRFA _(%)	21	39	19	31	39	47	62	61	10	68	39.8
	PE_PPFA _(%)	21	57	18	24	16	43	65	61	22	69	39.4
ANHM-01	PE_SRFA _(%)	20	7	62	53	54	54	55	45	23	17	38.8
	PE_PPFA _(%)	21	20	61	47	36	50	57	45	33	16	38.7
ANHM-02	PE_SRFA _(%)	520	147	87	94	94	96	83	98	1473	315	300.5
	PE_PPFA _(%)	519	152	87	93	92	95	84	98	1301	318	284.0
ANHM-03	PE_SRFA _(%)	193	61	97	94	87	91	85	90	343	47	118.7
	PE_PPFA _(%)	193	56	97	93	82	90	86	90	313	47	114.6
ANHM-04	PE_SRFA _(%)	279	232	91	91	98	98	97	96	18	481	158.1
	PE_PPFA _(%)	279	273	90	90	98	97	97	96	3	486	161.0
ANHM-05	PE_SRFA _(%)	44	87	80	68	83	86	93	93	21	116	76.9
	PE_PPFA _(%)	44	110	79	65	76	85	93	93	30	118	79.3
ANHM-06	PE_SRFA _(%)	205	305	97	96	97	99	99	97	127	551	177.3
	PE_PPFA _(%)	205	356	97	95	96	99	99	97	124	556	182.4

**Figure 2.**
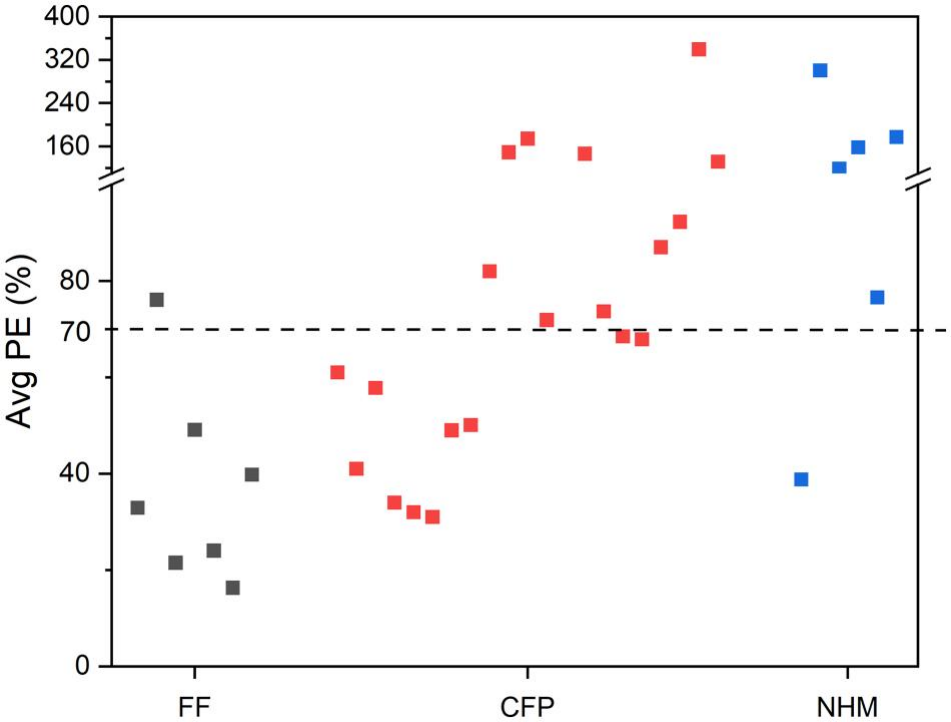
Average percent errors for Phase 1 FF, CFP, and NHM samples given a 70% fulvic cutoff.

The PE_avg_ values calculated for all 7 AFF, 21 ACFP, and 6 ANHM test samples analyzed in Phase 1 of the study are given in Figure 2. All but one of the FF samples had a PE_avg_ below 70%. On the other hand, all NHMs except for the molasses sample, ANHM-01, had a PE_avg_ greater than 70%. The PE_avg_ calculated for the CFP samples ranged from some having PE_avg_ values below 70%, while others had much higher PE_avg_ that were extremely high, like those typical of the ANHM test samples. Four of the ACFP test samples were also very close to PE_avg_ values of 70%.

### Influence of Metal Ions

Additional analysis for metal ion content was carried out to determine the degree of influence this had on the absorbance data and the calculated PE_avg_. The presence of metal ions may cause shifts in absorbance at wavelengths above 350 nm. Mingquan Yan in his works investigated the binding of various metal ions to FAs via UV-Vis spectroscopy (17, 18). The obtained differential spectra had four to six bands with maxima located at approximately 200, 240, 276, 316, 385, and 547 nm (17). Furthermore, the wavelength range from 350–400 nm was found to be indicative of the extent of metal-FA binding (18).

In standard SRFA, the total amount of metal is less than 0.3 ppm, which is expected, since the IHSS uses a series of desalting steps to remove most inorganic ions from their HFA standards. Of the 40 samples analyzed in Phase 1 of this study, 11 test samples had total metal ions concentration ranging between 5 and 14 ppm, while ACFP-20 had a total metal ion concentration of approximately 400 ppm. Thus, for these 12 samples the concentration of total metal ions was equal to or greater than the TOC concentration, which was 5 ppm. These samples included five out of the seven AFFs isolated from known humic ores.

To explore how the presence of metal ions in the test samples could affect the calculated PE_avg_, metal ions were added to the SRFA standard. Calcium, magnesium, and aluminum were chosen as the three metal ions to investigate. The metal ions were added individually or in combinations to the SRFA at concentrations ranging from 1 to 10 ppm. Table 5 provides the PE values between each of the test samples generated and the SRFA standard along with the calculated overall PE_avg_. The PE_avg_ ranged between 4 and 7% and interestingly did not correlate with an increase in the concentration of the metal ion, within the concentration range investigated. The PE_avg_ is primarily impacted by the change in absorbance at the higher wavelengths, impacting only three out of the ten indices in an appreciable way, namely absorbance at 355, 412, and 440 nm. Whether 1 ppm or 10 ppm of any one of the three metal ions were added, a PE of 10–17% was observed for absorbance at 412 and 440 nm. The main deviation in absorbance value with addition of metal ions were recorded at 412 and 440 nm. The obtained results agree with the work of Mingquan Yan (17) regarding the presence of metal ions impacting absorbance values at wavelengths greater than 350 nm. Most commercial fulvic products are FF and not HFA and would contain some metal ions. When test samples are prepared by diluting a product to a TOC concentration of 5 ppm, our data showed that it does not matter whether the metal ion concentration is 1 ppm or 10 ppm. The impact of the presence of metal ions on the calculated PE_avg_ is about 4–7%.

**Table 5.** MUVI percent errors based on the UV-Vis spectrum of SRFA with varying metal ion concentrations

Sample	S @ 275	S @ 350	a @ 254	a @ 280	a @ 325	a @ 355	a @ 412	a @ 440	SR(275/350)	a250/a365	PE_avg_
Al - 1 ppm	2.4	2.9	3.4	4	5.9	8.1	11.4	11.3	0.5	4.9	5.5
Al - 2.5 ppm	2.4	3.7	4.6	5.2	7.3	9.8	14.1	14.3	1.4	5.4	6.8
Al - 5 ppm	2.6	4.4	3.7	4.5	6.6	9.4	15.1	17.4	1.8	6	7.1
Al - 10 ppm	2.2	2.6	2.8	3.5	5.1	7.1	10	10.3	0.4	4.5	4.9
Ca - 1 ppm	2.9	4.2	2.5	3.2	5.5	7.8	13.1	14.3	1.3	5.5	6
Ca - 2.5 ppm	2.2	2.9	1.8	2.4	4.1	6.1	9.1	9	0.7	4.6	4.3
Ca - 5 ppm	2.9	4.7	1.9	2.6	4.8	7.2	12.9	14.3	2	5.7	5.9
Ca - 10 ppm	3	5.5	1.9	2.6	4.9	7.6	14.1	16.3	2.7	6.1	6.5
Ca 5 ppm + Al 5 ppm	2.1	3.5	2.6	3.2	5	7.4	11.2	11	1.4	5.2	5.3
Ca 5 ppm + Mg 5 ppm	2.9	4.8	1.6	2.2	4.4	7	12.2	13.3	2	6	5.6
Mg - 1 ppm	2.4	3	2.9	3.6	5.5	7.4	11	11.3	0.5	4.7	5.2
Mg - 2.5 ppm	2.7	3.4	2.9	3.7	5.6	7.6	11.4	11.3	0.7	5	5.4
Mg - 5 ppm	3	4.5	2.6	3.4	5.7	7.9	13.5	15.3	1.7	5.6	6.3
Mg -10 ppm	3.1	5.7	1.5	0.8	1.5	3.9	10.6	13.7	2.7	6.1	4.9
Mg 5 ppm + Al 5 ppm	2.9	4.3	2.2	2.9	4.9	7.6	12.4	13.3	1.4	5.7	5.8

### Influence of Carbon Concentration

**Figure 3.**
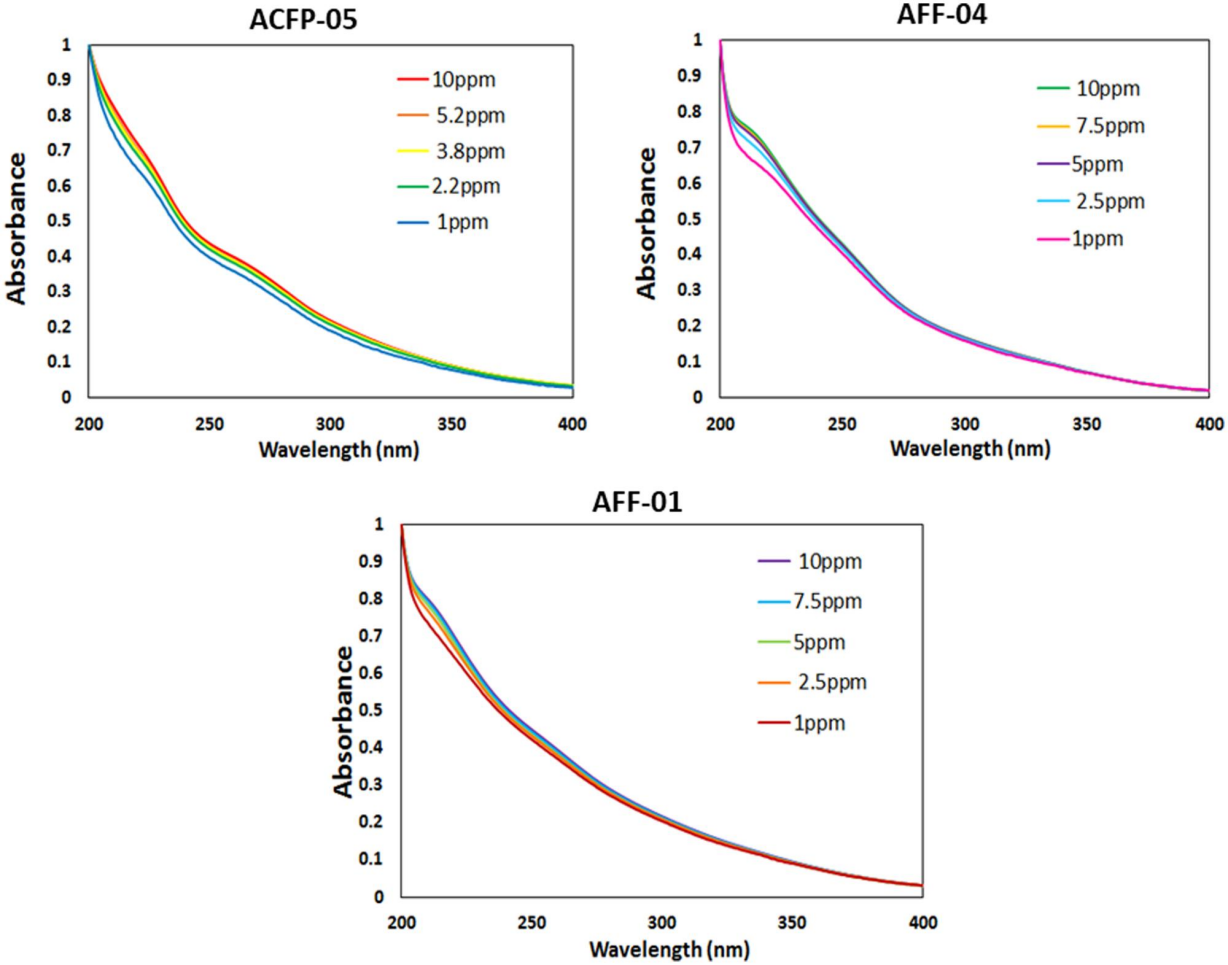
UV-Vis absorbance spectra for select Phase 1 samples at varying TOC concentrations.

In this study, samples with a TOC concentration of 5 ppm were utilized for UV-Vis analysis. However, for some samples, the absorbance signal (before normalization) was notably low at this concentration, indicating the likelihood of it being an adulterant product. At high concentrations it is possible that FA molecules will aggregate, causing deviations in the absorbance spectra. This was investigated by assessing the effect of concentration on the shape of UV-Vis spectra and the calculated parameters for three selected samples. Interestingly, there was no significant deviation observed in the shape of the UV spectra for samples within the concentration range of 1–10 ppm (Figure 3). This eliminated concerns over the accuracy of the TOC measurement and the dilution effect. This is expected since the normalization step of the calculations minimized the impact that concentration has on the PE_av_.

### Decision-Making Criteria and Threshold Values Proposed for the MUVI Method

**Figure 4.**
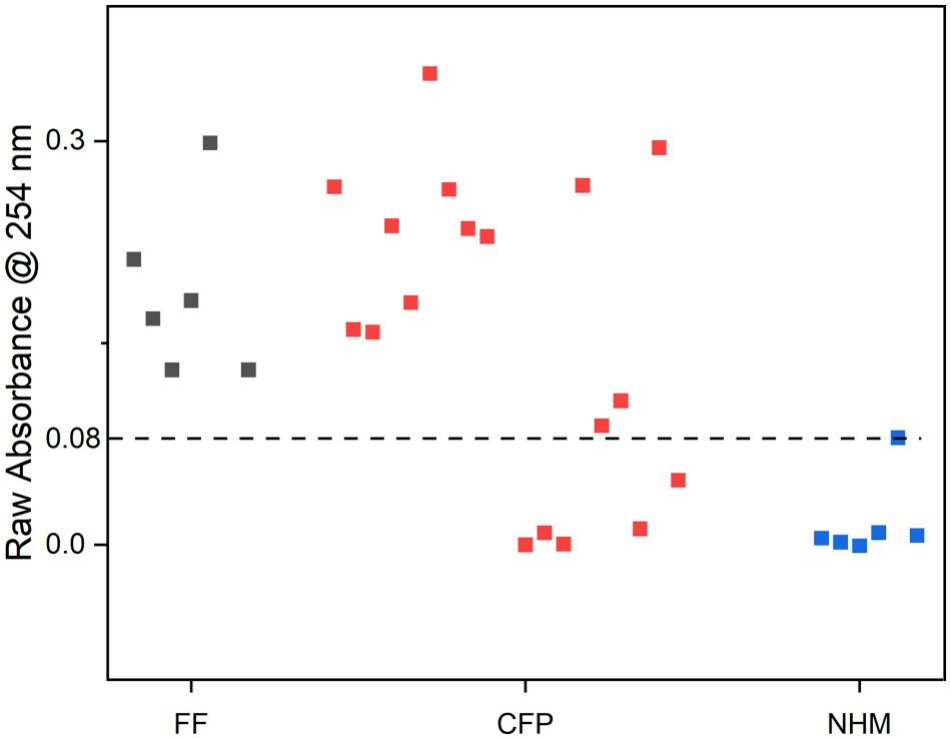
Raw UV-Vis absorbance values at 254 nm for Phase 1 FF, CFP, and NHM samples relative to a 0.08 absorbance fulvic cutoff. *Note*: The NHM with a raw absorbance near the 0.08 cutoff was determined as non-fulvic due to a very high PE_avg_ that was well above the 70% cutoff.

The main purpose of the proposed MUVI method was to develop an approach for testing products for presence of NHMs. All NHMs tested in the first phase, except for molasses, had PE_avg_ much greater than 70%. To address situations such as the one encountered with molasses, where the calculated PE_avg_ alone is insufficient for an accurate non-fulvic determination, it was necessary to identify an additional UV spectral feature characteristic of NHMs to be used in combination with the PE_avg_. Examining the raw (non-normalized) absorbances spectra at 254 nm, it was noticed that all the AFF test samples had values above 0.08, while most ANHM products had values less than 0.08. The raw absorbances measured for all the AFF, ACFP, and ANHM test samples characterized in Phase 1 of the study are shown in Figure 4.

Molasses (ANHM-01) was the only NHM tested with a calculated PE_avg_ below the 70% cutoff; however, molasses had a non-normalized absorbance at 254 nm wavelength of 0.005, which is well below the proposed 0.08 cutoff. Acetic acid (ANHM-02), citric acid (ANHM-03), corn steep liquor (ANHM-04), and the plant food protein (ANHM-06) all had both non-normalized absorbance much lower than 0.08 at 254 nm wavelength and calculated PE_avg_ much higher than 70%. The calcium lignosulfonate (ANHM-05) was the only one with a non-normalized absorbance at 254 nm of 0.08; however, the calculated PE_avg_ was 76.9%, placing it above the 70% cutoff. Thus, it was proposed to use the raw absorbance cutoff of >0.08 as an initial screening criterion for fulvic determination. When combined with a PE_avg_ cutoff value of 70%, all NHMs were accurately determined as non-fulvic.

The two cutoff parameters also proved accurate when applied to the FF fractions isolated from known HS (AFFs). The majority of the AFFs had non-normalized absorbance at 254 nm much greater than 0.08 and calculated PE_avg_ much lower than 70%. Table 6 summarizes the results for the non-normalized absorbance at 254 nm and calculated PE_avg_ for all 40 test samples analyzed in Phase 1. As the table indicates, there are some ACFP samples that had PE_avg_ values near the cutoff. For commercial products with PE_avg_ between 60 and 90%, it is therefore recommended to require additional analysis such as FT-ICR or C-NMR to support claims of a real FF. Thus, the method could be used for a substantial majority of products found in the market to make a simple binary decision. Results from this first phase of the study were promising as six of seven AFFs isolated from known humic ores were accurately determined as fulvic, while one of the samples had a slightly higher PE_avg_ value than the 70% cutoff. About half of the commercial products labelled as fulvic acid, 10 of 21, were determined as fulvic in this first phase. About five of these samples had PE_avg_ values that were close to the 70% cutoff. All six ANHM were determined as non-fulvic. Finally, all four AHSE test samples derived through alkaline extraction of Leonardite humic ores were accurately determined as non-fulvic, while the two ANOM samples obtained from IHSS were also accurately determined to be fulvic. The AHSE samples were derived from humified source material, but the FF of these samples was not isolated from the HA fraction, and as such most of the carbon in these samples belonged to HA structures rather than FAs. This is due to the much higher concentration of HA in the source material compared to FA. Using either the SRFA or the PPFA standards for comparison, these samples had PE_avg_ values ranging between 91 and 109%, which is well above the PE_avg_ of 70%.

**Table 6.** Average percent errors and fulvic classification summary for Phase 1 samples^a^

Sample name	a @ 254	Avg PE_SRFA _(%)	Avg PE_PPFA _(%)	Decision	Expected result
AFF-01	0.212	32.9	32.1	Fulvic	Yes
AFF-02	0.168	76.1	77.6	Non-fulvic	No
AFF-03	0.130	21.5	17.7	Fulvic	Yes
AFF-04	0.182	49.2	49.4	Fulvic	Yes
AFF-05	0.299	24.0	22.9	Fulvic	Yes
AFF-06	2.145	16.3	16.3	Fulvic	Yes
AFF-07	0.130	39.8	39.4	Fulvic	Yes
ACFP-01	0.266	61	62	Fulvic	NA
ACFP-02	0.160	41	40	Fulvic	NA
ACFP-03	0.158	58	59	Fulvic	NA
ACFP-04	0.237	34	33	Fulvic	NA
ACFP-05	0.180	32	29	Fulvic	NA
ACFP-06	0.350	31	31	Fulvic	NA
ACFP-07	0.264	49	49	Fulvic	NA
ACFP-08	0.235	50	51	Fulvic	NA
ACFP-09	0.229	82	91	Fulvic	NA
ACFP-10	0.522	149	159	Non-fulvic	NA
ACFP-11	0.000	174	165	Non-fulvic	NA
ACFP-12	0.009	71	68	Non-fulvic	NA
ACFP-13	0.000	945	841	Non-fulvic	NA
ACFP-14	0.267	146	146	Non-fulvic	NA
ACFP-15	0.088	74	71	Non-fulvic	NA
ACFP-16	0.107	68	64	Fulvic	NA
ACFP-17	0.012	68	67	Non-fulvic	NA
ACFP-18	0.295	87	84	Non-fulvic	NA
ACFP-19	0.048	92	91	Non-fulvic	NA
ACFP-20	1.479	339	335	Non-fulvic	NA
ACFP-21	3.019	131	129	Non-fulvic	NA
ANHM-01	0.005	38.8	38.7	Non-fulvic	Yes
ANHM-02	0.002	300.5	284.0	Non-fulvic	Yes
ANHM-03	0.000	118.7	114.6	Non-fulvic	Yes
ANHM-04	0.009	158.1	161.0	Non-fulvic	Yes
ANHM-05	0.080	76.9	79.3	Non-fulvic	Yes
ANHM-06	0.007	177.3	182.4	Non-fulvic	Yes
AHSE-01	0.529	102.1	109.2	Non-fulvic	Yes
AHSE-02	0.500	95.5	102.9	Non-fulvic	Yes
AHSE-03	0.915	98.6	105.2	Non-fulvic	Yes
AHSE-04	0.807	91.3	99.2%	Non-fulvic	Yes
ANOM-01	0.586	28.0	ND	Fulvic	Yes
ANOM-02	0.221	6.1	ND	Fulvic	Yes

a Yes = Correct identification; No = False-negative or false-positive result; ND = Not Determined (only tested against SRFA); NA = Not Applicable (for commercial fuvlic products where the source material is unknown).

### Phase 2—MUVI Method Results

A total of nine samples from Phase 1 including four AFF, four ACFP and one ANOM test samples were analyzed. Fourteen new samples (Table 3) were also analyzed consisting of four BFF, five BCFP (commercial fulvic product), four BNHM and one BHFA. Results for all 23 samples analyzed in this phase are given in Table 7. All four AFF samples were determined as fulvic. It should be noted that in Phase 1 of the study AFF-02 (FF isolated from New Mexico ore) was the only FF isolated from a known humic ore that failed with calculated PE_avg_ of 76% (PE_SRFA_) and 78% (PE_PPFA_). In Phase 2 of the study the same sample resulted in PE_avg_ of 49% (PE_SRFA_) and 44% (PE_PPFA_), which is well below the 70% cutoff. There was agreement using the MUVI method in all the other eight samples that were tested by both laboratories, including the four commercial products (Table 7).

**Table 7.** Average percent errors and fulvic classification summary for Phase 1 repeat samples and Phase 2 samples^a^

Sample name	a @ 254	Avg PE_SRFA_ (%)	Avg PE_PPFA_ (%)	Decision	Expected result
AFF-01	0.247	40.5	41.4	Fulvic	Yes
AFF-02	0.327	49.3	43.8	Fulvic	Yes
AFF-03	0.175	31.3	24.0	Fulvic	Yes
AFF-04	0.407	31.3	27.7	Fulvic	Yes
ACFP-01	0.435	42.2	39.5	Fulvic	NA
ACFP-02	0.265	25.0	25.0	Fulvic	NA
ACFP-03	0.207	45.9	44.4	Fulvic	NA
ACFP-04	0.266	33.2	32.0	Fulvic	NA
ANOM-01	0.140	30.0	30.1	Fulvic	Yes
BFF-01	0.247	30.5	20.2	Fulvic	Yes
BFF-02	0.191	16.7	18.9	Fulvic	Yes
BFF-03	0.173	40.7	38.6	Fulvic	Yes
BFF-04	0.201	57.0	57.7	Fulvic	Yes
BCFP-01	0.212	47.1	47.6	Fulvic	NA
BCFP-02	0.175	40.9	38.8	Fulvic	NA
BCFP-03	0.180	46.9	47.5	Fulvic	NA
BCFP-04	0.120	46.5	47.1	Fulvic	NA
BCFP-05	0.351	26.3	27.6	Fulvic	NA
BHFA-01	0.175	20.6	20.4	Fulvic	Yes
BNHM-01	0.045	94.7	86.2	Non-fulvic	Yes
BNHM-02	0.074	136.3	123.2	Non-fulvic	Yes
BNHM-03	0.072	29.7	30.0	Non-fulvic	Yes
BNHM-04	0.056	121.0	107.3	Non-fulvic	Yes

a Yes = Correct identification; No = False-negative or false-positive result; ND = Not Determined (only tested against SRFA); NA = Not Applicable (for commercial fuvlic products where the source material is unknown).

Furthermore, using the MUVI method the four BFFs, which included a N.D Leonardite FF, Canadian sphagnum peat FF, Irish moss peat FF, and Balkan black peat FF test samples, were accurately determined as fulvic, while all four BNHMs, which included two seaweed extracts, a sugarcane molasses and a yucca extract, were accurately determined as non-fulvic. Finally, the one BHFA test sample consisting of IHSS Elliott Soil FA Standard V was also accurately determined as fulvic.

### Proposed MUVI Method Procedures

The results from this study demonstrate promising potential for the MUVI method being used as a relatively simple and easy screening method to determine if a commercial product labelled as a FA is likely to contain FFs. The MUVI method procedures are provided in Figure 5. The details for each step in the method are provided below.

- *Preparing test samples for analysis.—*
  1. *For dry/solid products.—*Crush a representative portion of the product into a fine homogeneous powder and screen through a 75 µm sieve. Weigh out a 1.00 ± 0.05 g test portion of the sieved material and transfer it to a 1 L Erlenmeyer flask. Add DI water, while constantly stirring on a stir-plate, to a final volume of 1 L. Stir the liquid sample for approximately 30 min. Fulvic powder should be readily soluble in DI water; however, if a sizable portion remains insoluble, increase the stirring time up to 4 h.
  2. *For liquid products.—*Thoroughly shake/stir the content of product in its original container for approximately 1 min. Weigh out a 1.00 ± 0.05 g test portion of the liquid and transfer it to a 1 L Erlenmeyer flask. Add DI water, while constantly stirring on a stir-plate, to a final volume of 1 L. Stir the liquid sample for approximately 30 min.
*Note*: From this point on, test portions prepared from either liquid or dry/solid product samples follow the same method.
- *Separation of insoluble fraction.—*Transfer the 1 L test portion to centrifuge tubes and centrifuge the entire volume at 3900 × *g* for 30 min to separate any insoluble material that may have contaminated the sample. Decant the soluble fraction from the centrifuge tubes into a 1 L Erlenmeyer flask and discard the insoluble material.
- *Acid precipitation of HA fraction.—*Adjust the pH of the soluble fraction from step **(b)** by slowly adding 6M HCl while gently stirring the solution until a final pH of 1.0 ± 0.1 is reached. Cover the flask with parafilm and mix for 1 h.
- *Separation of HA fraction.—*Allow the pH-adjusted solution to sit undisturbed for 4 h, enabling the HA fraction to flocculate and drop out of solution. Immediately centrifuge the entire volume at 3900 × *g* for 30 min using pre-weighed 50 mL high-temperature centrifuge tubes allowing the recovery of the separated HA fraction. Decant the solution which contains the FF into a 1 L Erlenmeyer flask. Centrifuge the tubes containing the insoluble flocculated HA fraction again at 1500 × *g* for 30 min to further separate the FF solution from the HA fraction. Decant any additional supernatant recovered from this step into the solution recovered from the first stage of HA precipitate centrifugation.
- *Quantification of product HA fraction.—*If a sizeable quantity of HA fraction is recovered through step (**d**), and there is a desire to quantify this content, use the method outlined in sections 9.2 and 10 of ISO 19822 (2018) to quantify the HA content (26). The steps consist of transferring the pre-weighed high-temperature centrifuge tubes containing the HA fraction to a drying oven at 62 ± 3°C. Break up any clumps that formed during drying using a glass rod and allow to dry for at least 24 h in the oven. Once the flocculated HA fraction is dried to a constant weight, transfer the tubes to a desiccator to cool to room temperature. Once cooled, record the combined weight and subtract from the pre-weight of the tube to obtain the weight of the HA fraction. At this stage, the HA fraction will also contain some ash content.
  Determine the residual ash content by transferring and combining all the HA flocculate recovered from the tubes to a crucible that has been washed in acetone and dried in a muffle furnace for 2 h at 500°C. The crucible should be cooled to room temperature using a desiccator prior to having the HA fraction transferred to it. Ash the sample in a muffle furnace for 4 h at 500°C until constant weight is achieved. Remove the crucible from the muffle furnace and transfer to a desiccator and let the crucible cool down to room temperature. Weigh the crucible and obtain the weight of HA by subtracting the ash weight from the dry weight.
- *Measurement of the Fulvic Fraction TOC.—*Take a portion of the supernatant from (**d**) which contains the FF and measure the TOC of the test portion in triplicate using either the combustion catalytic oxidation (CCO) or the persulfate oxidation method. If the three results are in close agreement, average the triplicate TOC values and use in subsequent calculations. If not in close agreement, make one or two additional measurements of TOC to determine the outlying value and obtain an average TOC value.
- *Preparing 5 ppm C test solutions for UV-absorbance analysis.—*Transfer to test tubes small portions of the FF from **(d)** and based on the average TOC measurements from **(f)** dilute with DI water to generate 10 mL test solutions with 5 ppm C in triplicate for use in obtaining UV absorbance.
- *Preparing the SRFA and PPFA standards.—*The IHSS SRFA and PPFA standards are purified HFA fractions that have been isolated using a series of desalting steps that result in very low ash content. Weigh out 1.0 mg test portion of each standard and transfer to a 50 mL Erlenmeyer flask. Add DI water while constantly stirring to a final volume of 20 mL. Stir the liquid sample for approximately 30 min. Take three portions of the solution and measure the TOC in triplicate and calculate an average TOC. Transfer to test tubes small portions of the standards and dilute with DI water to generate 10 mL test solutions with 5 ppm C in triplicates for use in obtaining UV absorbance.
- *UV-Absorbance data analysis and calculations.—*
  1. Make sure the spectrophotometer has been calibrated using a reference cell (a holmium oxide reference cell was used in this study).
  2. Prepare a buffer baseline solution by slowly adding 6M HCl to DI water while gently stirring the solution until a final pH of 1.0 ± 0.1 is reached. Transfer an aliquot of the baseline buffer solution to a 1 cm quartz cuvette and obtain the absorbance values between wavelengths of 200 and 800 nm. Repeat the transfer of the aliquot and the acquisition of absorbance two more times to obtain UV data for the buffer solution in triplicate.
  3. Transfer aliquots from the 5 ppm C test portions prepared for the standards (SRFA and PPFA) and products to a 1 cm quartz cuvette and obtain absorbance data (200–800 nm) in triplicate.
  4. Export all absorbance data into an excel file and observe the raw absorbance at 254 nm. Any sample with a raw (non-normalized) absorbance value of <0.08 at 254 nm would fail this method due to the extremely low absorbance at 254 nm and would be designated as non-fulvic.
  5. Normalize all UV absorbance data obtained by dividing the absorbance at each wavelength between 200 and 800 nm by the maximum absorbance for that test portion.
  6. Copy the normalized absorbance data into ASFit and obtain a calculated value for each of the 10 indices outlined in Table 1. (Alternatively, you can also manually calculate each index from the normalized data.)
  7. Calculate the percent error (PE) between the sample and both the SRFA and PPFA standards for each index value using
    $$\%\;\mathrm{Error}=|\frac{v_{a}-v_{s}}{v_{s}}|*100$$
  8. Ensure that the three replicates have PE values that agree with each other across all 10 indices. If there are greater than 20% differences found, repeat the acquisition of the UV-Vis with a new aliquot to ensure that the three replicates are in reasonable alignment.
  9. Average the PE across the triplicate data obtained for each product.
  10. Calculate the equal-weighted overall average percent error (PE_avg_) for that sample and if the PE_avg_ is greater than 70%, the sample is designated as non-fulvic using the method.
- *Decision-making process using the MUVI method.—*The combination of two criteria from the data analysis are used to make a binary decision on a product tested using the MUVI method.
  1. Non-normalized absorbance at 254 nm wavelength greater than 0.08.
  2. Calculated PE_avg_ value of less than 70%.

It is important to note that the PE_avg_ calculations comprise 10 distinct features of the UV-absorbance curve of SRFA or PPFA, thereby providing a much more robust analysis than relying on any specific index or range of wavelengths. In most products, where both criteria are satisfied, an accurate fulvic determination can be made. If either one of these criteria are not met, a non-fulvic determination is made. For products where the calculated PE_avg_ value is in the range from 60 to 90%, additional FTICR or C-NMR data may be required. Thus, the method can be used for the majority of products, while a small fraction may require the additional FTICR or C-NMR data.

## Conclusions

FAs are playing a growing and prominent role as biostimulants in improving the sustainability of global food production. HS are extremely heterogeneous, consisting of complex mixtures of thousands of different molecular structures. Many plant extracts and effluents from industrial activities are also loaded with heterogeneous mixtures of organic molecules and, as such, can mimic some of the physical, chemical, and even functional properties of FAs.

In this study, we propose a simple method of leveraging multiple UV absorbance spectral features of HFA to differentiate genuine fulvic products from adulterants. At the core of the proposed MUVI method is the evaluation of the percent error for 10 different indices (Table 1) that constitute important features of the UV absorbance curve of HS. A total of 54 test samples were analyzed across two phases of the study with 9 samples being analyzed in both phases by two different laboratories.

Results obtained for the proposed MUVI method were promising, with all 11 FFs isolated from known HS (Phases 1 and 2) accurately determined as FF, and all 11 non-humified materials accurately determined as non-fulvic. A total of 25 commercial products labelled as a FA were also studied and 14 of the 25 samples were found to be fulvic, while 11 were found to be non-fulvic. All four AHSE test samples derived by alkali extraction of Leonardite humic ores were determined as non-fulvic. This is promising, as it indicates that the MUVI method may be applicable to distinguish products containing HA from products containing FF. The potential for using the MUVI method to distinguish between HA and FF requires further study and validation.

Additional analysis for metal ion content showed that there was no correlation observed between PE_avg_ and increased metal ion concentration in the range investigated. The effect of test portion concentration on the shape of UV-Vis spectra was investigated for three selected samples and there was no significant deviation observed in the shape of the UV spectra for samples within the concentration range of 1–10 ppm.

## CRediT Author Statement

Elena Vialykh (Conceptualization [Equal], Data curation [Equal], Formal analysis [Equal], Investigation [Equal], Methodology [Equal], Writing—original draft [Lead], Writing—review & editing [Supporting]), Shelby Buckley (Conceptualization [Equal], Data curation [Equal], Formal analysis [Equal], Investigation [Equal], Methodology [Equal], Writing—original draft [Equal], Writing—review & editing [Equal]), Julia Gentile (Investigation [Equal]), Mohammad Rahbari (Conceptualization [Equal], Data curation [Lead], Formal analysis [Lead], Funding acquisition [Lead], Methodology [Equal], Supervision [Lead], Writing—review & editing [Lead]), Fernando Rosario-Ortiz (Conceptualization [Lead], Funding acquisition [Lead], Supervision [Lead]), Richard Lamar (Investigation [Equal], Writing—review & editing [Equal]), and Jarrod Psutka (Data curation [Equal], Formal analysis [Equal], Investigation [Equal], Methodology [Equal], Validation [Equal], Writing—review & editing [Equal])
